# Quercetin metabolism by fecal microbiota from healthy elderly human subjects

**DOI:** 10.1371/journal.pone.0188271

**Published:** 2017-11-27

**Authors:** Motoi Tamura, Chigusa Hoshi, Masuko Kobori, Shunsuke Takahashi, Junko Tomita, Mie Nishimura, Jun Nishihira

**Affiliations:** 1 Food Research Institute of National Agriculture and Food Research Organization, Tsukuba, Ibaraki, Japan; 2 TechnoSuruga Laboratory Co., Ltd., Shizuoka-shi, Shizuoka, Japan; 3 Department of Medical Management and Informatics, Hokkaido Information University, Ebetsu, Hokkaido, Japan; University of Illinois at Urbana-Champaign, UNITED STATES

## Abstract

Quercetin is a polyphenol found in food that has numerous health benefits. This study investigated the relationship between quercetin metabolism, gut microbiota composition, and dietary intake in elderly Japanese subjects. A food frequency questionnaire was used to assess dietary intake during the week prior to stool sample collection. Fecal suspensions from 56 subjects were anaerobically incubated with quercetin and fecal microbiota composition was analyzed by next-generation sequencing. Inter-individual variations in quercetin concentration and fecal microbiota composition at family level suggested differences in microbial quercetin metabolism. The abundance of *Sutterellaceae* (r = −0.292) and *Oscillospiraceae* (r = −0.334) was negatively correlated whereas that of *Fusobacteriaceae* (r = 0.361) and *Enterobacteriaceae* (r = 0.321) was positively correlated with quercetin concentration. Niacin (r = −0.313), vitamin B_6_ (r = −0.297), vitamin B_12_ (r = −0.266), vitamin D (r = −0.301), and ratio of animal protein to total protein (r = −0.27) were also negatively correlated with quercetin concentration. Bacterial abundance was positively or negatively related to intake of food components. This is the first report describing the relationship between fecal quercetin metabolism, human microbiota, and dietary intake in the elderly.

## Introduction

Quercetin is a polyphenol found in plants that has health benefits. Studies in mice have shown that chronic dietary intake of quercetin alleviates hepatic fat accumulation [1] and cardiovascular diseases [2]. In addition, quercetin has been reported to prevent hepatic cancer [3] and azoxymethane-induced colorectal carcinogenesis [4] in rats.

The effects of quercetin in humans have been extensively studied. In healthy male smokers, daily intake of quercetin from onion peel extract improved blood lipid profiles, glucose, and pressure [5]. Quercetin exhibited blood pressure-lowering effects in overweight/obese subjects with the apo epsilon3/epsilon3 genotype [6], and reduced blood pressure in hypertensive subjects [7].

Intestinal microbiota metabolize various polyphenols, including quercetin; the human intestinal bacterium *Eubacterium ramulus* has also been reported to degrade quercetin and luteolin [8]. Anaerobic degradation of quercetin by *Clostridium orbiscindens* [9] and fecal microbial metabolism of quercetin have also been reported [10]. Thus, rapid biotransformation of quercetin by intestinal microbiota alters quercetin bioavailability in the lower gut.

Several recent studies have investigated the relationship between intestinal flora and obesity. In humans, obesity was found to be associated with changes in the relative abundance of Bacteroidetes and the Firmicutes, the two predominant phyla [11]. Diet (i.e., nutrient load) can also affect gut bacterial community structure [12,13]. These findings highlight the importance of diet on microbiota composition; however, it is unclear how quercetin metabolism is affected by intestinal microbiota. To address this issue, in this study we investigated the relationships among dietary intake and metabolism by intestinal microbiota and gut microbial community structure in elderly Japanese subjects.

## Materials and methods

### Chemicals

Quercetin were purchased from Funakoshi (Tokyo, Japan).

### Study subjects

To identify nutritional factors affecting quercetin and daidzein metabolism by intestinal microbiota, we recruited 87 healthy volunteers by advertisements. We screened all subjects and excluded individuals 1) receiving medications for dementia, Alzheimer's disease, psychiatric disorders, or cerebrovascular diseases; 2) receiving hormone therapy; 3) with a history of psychiatric disorders, cerebrovascular diseases, or gastrointestinal disorders; 4) with severe acute or chronic diseases; 5) who underwent surgery; or 6) with a severe allergic reaction to food. We selected healthy male (n = 31; mean age: 71 ± 0.7 years, range: 65–78 years) and female (n = 25; mean age: 73.5 ± 1.0 years; range: 65–84 years) subjects. Participants were asked to fill out a food frequency questionnaire based on food groups (FFQg) regarding their dietary intake for 1 week prior to stool sample collection. BMI was calculated based on self-reported height and weight. The study was performed in accordance with the principles of the Declaration of Helsinki. Subjects provided written, informed consent for their participation in the study. The study protocol was approved by the Human Investigations Review Board of the National Food Research Institute (approval date: April 7, 2014; approval number: HU2014-07a) and Hokkaido Information University (approval date: Dec 1, 2014; approval number: 2014–19). The study was registered with the University Hospital Medical Information Network (approval number: UMIN000015940).

### Nutritional intake

The food frequency questionnaire based on food groups (FFQg)[14] was answered by all participants and used to calculate dietary intake for the week prior to stool sample collection. The FFQg was based on 29 food groups and 10 modes of cooking in commonly used units or portion sizes. The validity of FFQg was verified compared with the 7-day meal recording method for 66 Japanese subjects. The ratio between FFQg and meal recording method was between 90 and 110%, and the average of all nutrients was 104% [14]. This FFQg can evaluate diet intake and frequency within one week. Energy and nutrition intake was estimated for each participant from FFQg data using Excel Eiyoukun v.2.0 software (Yoshimura Y and Takahashi K, Kenpakusha, Tokyo, Japan), which was designed to calculate amounts of ingredients on the fifth edition of the Standard Tables of Food Composition in Japan. Intake of energy, and macronutrients and micronutrients was assessed by this program. We determined daily energy intake and obtained measures of protein (g/day), fat (g/day), carbohydrate (g/day), ash (g/day), water (g/day), saturated fatty acid (g/day), monounsaturated fatty acid (g/day), polyunsaturated fatty acid (g/day), n-3 polyunsaturated fatty acid (g/day), n-6 polyunsaturated fatty acid (g/day), cholesterol (mg/day), soluble dietary fiber (g/day), insoluble dietary fiber (g/day), total dietary fiber (g/day), retinol (μg/day), α-carotene (μg/day), β-carotene (μg/day), β-carotene equivalent (μg/day), retinol (μg/day), retinol equivalent (μg/day), vitamin D (μg/day), α-tocopherol (mg/day), vitamin K (μg/day), vitamin B_1_ (mg/day), vitamin B_2_ (mg/day), niacin (mg/day), niacin equivalent (mg/day), vitamin B_6_ (mg/day), vitamin B_12_ (μg/day), folic acid (μg/day), pantothenic acid (mg/day), vitamin C (mg/day), biotin (μg/day), K (mg/day), Na (mg/day), Ca (mg/day), Mg (mg/day), P (mg/day), Fe (mg/day), Zn (mg/day), Cu (mg/day), Mn (mg/day), I (μg/day), Se (μg/day), Cr (μg/day), Mo (μg/day), NaCl (g/day), ethanol (g/day), protein energy ratio (%), fat energy ratio (%), saturated fatty acids energy ratio (%), carbohydrate energy ratio (%), alcohol energy ratio (%), cereals energy ratio (%), animal protein ratio (%), and green and yellow vegetables ratio (%).

### Stool sampling and analysis

Stool samples were collected on paper sheets and quickly transferred to sterilized containers (Sarstedt K.K., Tokyo, Japan) that were placed in an AnaeroPouch with a CO_2_ generator (Mitsubishi Gas Chemical Company), Tokyo, Japan and transported to the National Food Research Institute by parcel delivery service with the temperature maintained below 10°C. Approximately 0.1 g of stools was transferred to a sterilized glass homogenizer to which 30-fold anaerobic medium was added, followed by homogenization by gassing with O_2_-free CO_2_. The anaerobic medium was prepared as follows: brain heart infusion (37 g), agar (1 g), L-cysteine HCl·H_2_O (0.5 g), and Na_2_CO_3_ (4 g) were dissolved in 1000 ml distilled water. Aliquots of the broth (9 ml) were transferred to test tubes that were gassed with O_2_-free CO_2_, sealed with a butyl rubber stopper, and sterilized by autoclaving. Quercetin (20 mg) was dissolved in 1 ml dimethyl sulfoxide. The quercetin solution (2 μl) was combined with 0.2 ml of homogenate and the mixture was incubated under a CO_2_ atmosphere generated using the AnaeroPack system (Mitsubishi Gas Chemical Company for 7 or 24 h at 37°C. Methanol-acetic acid (100:5, v/v) was added to the reaction mixture to a total volume of 1.0 ml. The mixture was vortexed for 120 s and centrifuged at 11,000 × g and 4°C for 10 min. The supernatant was analyzed by high-performance liquid chromatography (HPLC) as follows: 20 μl sample were injected into a 250 × 4.6 mm Capcell Pak C18 5 μm column (Shiseido, Tokyo, Japan). A Jasco MD-2018 photodiode array detector (Jasco Co., Tokyo, Japan) was used to detect quercetin by spectral analysis from 200–400 nm for each peak. Spectral data at 254 nm were used to quantify quercetin content, with pure quercetin used as a standard. The mobile phase consisted of methanol/acetic acid/water (35:5:60, v/v/v). The HPLC system was operated at a column temperature of 40°C and a flow rate of 1 ml/min.

### DNA extraction from stool samples

DNA was extracted from stool samples as previously described [15]. Stool samples were resuspended in a buffer containing 4 M guanidium thiocyanate, 100 mM Tris-HCl (pH 9.0), and 40 mM EDTA and mixed with zirconia beads using the FastPrep FP100A instrument (MP Biomedicals, Irvine, CA, USA). DNA was extracted using a Magtration System 12GC and GC series MagDEA DNA 200 reaction cartridge (Precision System Science, Tokyo, Japan). The final concentration of the DNA sample was adjusted to 10 ng/μl.

### Analysis of human fecal microbiota by next-generation sequencing (NGS)

Human fecal bacteria 16S rRNA was analyzed by NGS using the MiSeq system (Illumina, San Diego, CA, USA) as previously described [15]. The V3–V4 hypervariable regions of 16S rRNA were PCR amplified from microbial genomic DNA using prokaryote universal primers (Pro341F/Pro805R) [15] and the dual-index method [16]. Barcoded amplicons were sequenced using the paired-end, 2 × 284-bp cycle run on the MiSeq system with MiSeq Reagent kit v.3 (600 Cycles).

### Bioinformatics analysis

Bioinformatics analysis was performed as previously described [15]. Overlapping paired-end reads were merged using the fastq-join program with default settings [17]. The reads were processed with quality and chimera filtering as follows: only reads with a quality value score of 20 for > 99% of sequences were extracted, and chimeric sequences were removed using the usearch6.1 tool [18]. Non-chimeric reads were submitted for 16S rDNA-based taxonomic analysis using the Ribosomal Database Project Multiclassifier tool [19]. Reads obtained in the Multi-FASTA format were assigned at phylum and genus levels with an 80% confidence threshold.

### Statistical analysis

Data are expressed as mean ± standard error and were analyzed using Sigma Plot v.11 (Systat Software, San Jose, CA, USA). Differences between groups were compared with the Spearman rank order correlation tests. A P value < 0.05 was considered statistically significant.

## Results

### Characteristics of study subjects

The age range of participants was 65–84 years (mean ± standard error, 72.1 ± 0.6 years); the mean height ± standard error was 158.6 ± 1.2 cm; mean body weight ± standard error was 58.7 ± 1.5 kg; and mean body mass index (BMI) ± standard error was 23.1 ± 0.4.

### FFQg results

The results of the FFQg are shown in Table 1. Mean intake per day of energy, carbohydrate, protein, and lipid was 1895.49 ± 56.02 kcal, 254.2 ± 6.73 g, 66.69 ± 1.79 g, and 59.79 ± 2.21 g, respectively. Daily protein intake was higher than daily lipid intake. Carbohydrate, lipid, and protein energy ratios were 57.55% ± 0.73%, 28.26% ± 0.6%, and 14.18% ± 0.23%, respectively.

**Table 1:** Daily dietary intake in elderly Japanese subjects according to FFQg results.

	n = 56		
Energy(K cal)	1895.49	±	56.02
Water (g/d)	1046.88	±	38.96
Proetein (g/d)	66.69	±	1.79
Fat (g/d)	59.79	±	2.21
Carbohydrate (g/d)	254.20	±	6.73
Ash (g/d)	17.69	±	0.51
Na (mg/d)	4084.26	±	142.13
K (mg/d)	2405.13	±	68.73
Ca (mg/d)	596.34	±	24.64
Mg (mg/d)	249.81	±	7.58
P (mg/d)	1028.34	±	30.57
Fe (mg/d)	7.42	±	0.24
Zn (mg/d)	7.60	±	0.20
Cu (mg/d)	1.06	±	0.03
Mn (mg/d)	2.57	±	0.07
Iodine (μg/d)	837.48	±	116.01
Se (μg/d)	53.06	±	1.77
Cr (μg/d)	7.08	±	0.28
Mo (μg/d)	140.30	±	5.08
Retinol (μg/d)	188.17	±	8.16
β-Caroten (μg/d)	2704.98	±	164.30
β-Caroten equivalent (μg/d)	3449.15	±	191.69
Retinol equivalent (μg/d)	486.08	±	20.26
Vitamin D (μg/d)	6.63	±	0.33
αTocophrol (mg/d)	6.45	±	0.23
Vitamin K (μg/d)	193.81	±	7.90
Vitamin B_1_ (mg/d)	0.91	±	0.03
Vitamin B_2_ (mg/d)	1.07	±	0.03
Naiacin (mg/d)	14.77	±	0.50
Naiacin equivalent (mg/d)	27.81	±	0.81
Vitamin B_6_ (mg/d)	1.16	±	0.04
Vitamin B_12_ (μg/d)	6.83	±	0.33
Folic acid (μg/d)	284.27	±	8.44
Pantothenic acid (mg/d)	5.35	±	0.14
Biotin (μg/d)	29.84	±	1.04
Vitamin C (mg/d)	118.77	±	5.15
Saturated fatty acids (g/d)	18.22	±	0.73
Monounsaturated fattyacids (g/d)	20.34	±	0.81
Polyunsaturated faty acids (g/d)	12.47	±	0.53
Cholesterol (μg/d)	280.28	±	12.56
Soluble fiber (g/d)	3.55	±	0.11
Insoluble fiber (g/d)	10.69	±	0.31
Total fiber (g/d)	14.80	±	0.43
NaCl (g/d)	10.35	±	0.36
Ethanol (g/d)	8.92	±	2.31
Total Fat (g/d)	51.15	±	1.94
n-3 fatty acids (g/d)	2.36	±	0.10
n-6 fatty acids (g/d)	10.08	±	0.44
Protein energy ratio (%)	14.18	±	0.23
Fat energy ratio (%)	28.26	±	0.60
Saturated fatty acids energy ratio (%)	8.61	±	0.22
Carbohydrate energy ratio (%)	57.55	±	0.73
Alcohol energy ratio (%)	2.72	±	0.66
Cereals energy ratio (%)	33.15	±	1.09
Animal protein ratio (%)	51.21	±	1.06
Green and yellow vegetables ratio (%)	25.58	±	1.42

### Quercetin metabolism by fecal microbiota

**Fig 1:**
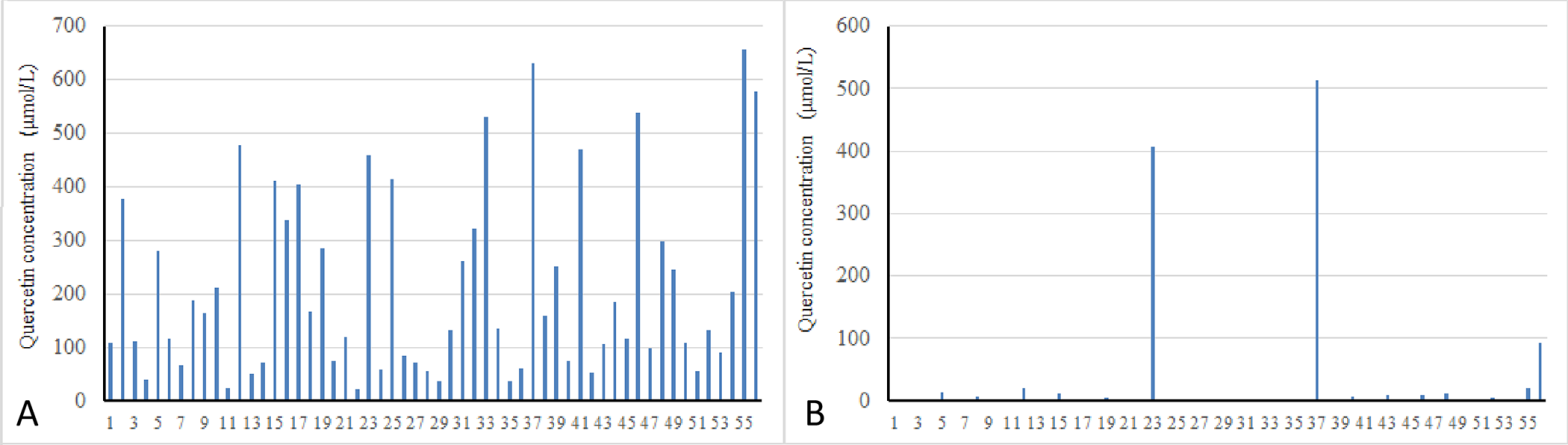
**Quercetin concentration in fecal samples (A, B).** Quercetin was anaerobically incubated with fecal suspensions for 7 h (A) or 24 h (B). N = 56. The X axis number indicates the volunteer number.

Anaerobic incubation of fecal suspensions with quercetin for 7 h revealed inter-individual variations in quercetin concentration, suggesting a difference in microbial metabolism of quercetin (Fig 1A). This variation disappeared after incubation for 24 h as a result of quercetin degradation (Fig 1B).

### Fecal microbiota composition

**Fig 2:**
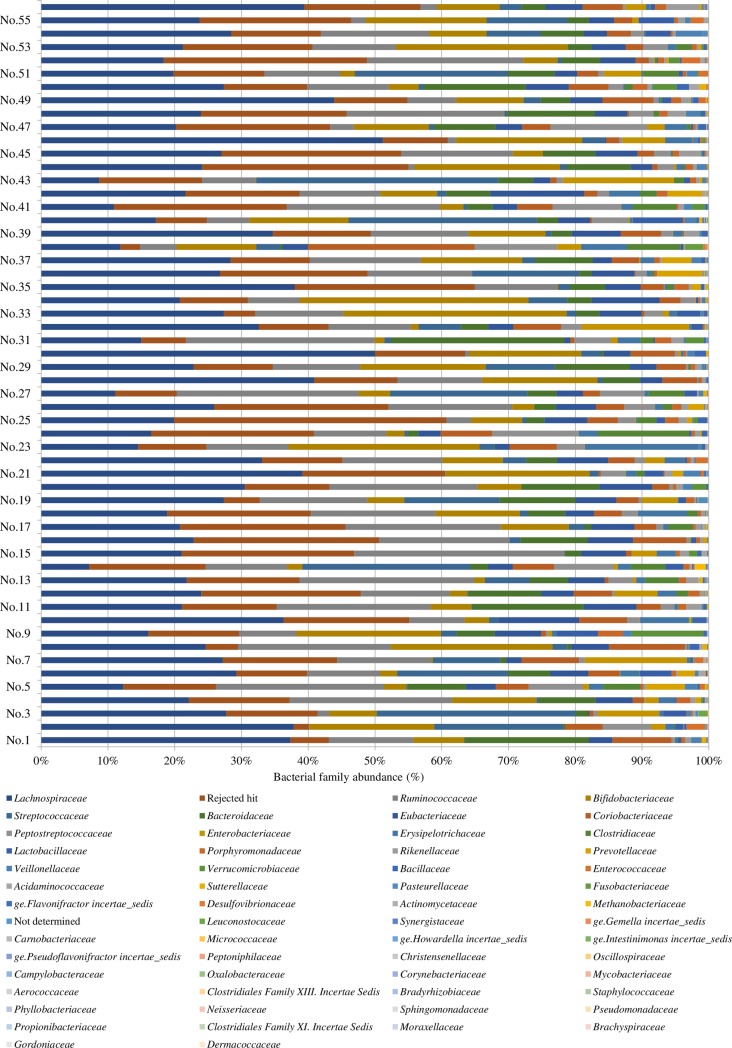
Fecal microbiota composition at the family level in samples from elderly Japanese subjects. Columns are color-coded according to bacterial families. The X axis shows the bacterial family abundance (%). The Y axis shows the volunteer number.

The microbial community structure of stool samples is shown in Fig 2. The most abundant phyla were *Lachnospiraceae* (25.4% ± 1.3%), *Ruminococcaceae* (13.5% ± 1.0%), *Bifidobacteriaceae* (9.9% ± 1.2%), *Streptococcaceae* (6.0% ± 1.2%), *Bacteroidaceae* (5.9% ± 0.7%), *Eubacteriaceae* (4.9% ± 0.4%), *Coriobacteriaceae* (4.3% ± 0.5%), *Peptostreptococcaceae* (2.8% ± 0.5%), *Enterobacteriaceae* (2.0% ± 0.5%), *Erysipelotrichaceae* (1.7% ± 0.4%), *Clostridiaceae* (1.5% ± 0.3%), *Lactobacillaceae* (1.0% ± 0.2%), *Porphyromonadaceae* (0.8% ± 0.1%), *Rikenellaceae* (0.7% ± 0.1%), and *Prevotellaceae* (0.6% ± 0.2%). Inter-individual variation in fecal microbiota composition at family level was observed.

### Correlation between FFQg data and fecal microbiota composition and quercetin concentration

Given the lack of inter-individual variation in quercetin concentrations following anaerobic incubation of fecal suspensions with quercetin for 24 h, we analyzed the relationship between quercetin concentration and FFQg data (intake of energy, and macronutrients and micronutrients) and fecal microbiota composition after a 7-h incubation period. There were significant correlations between FFQg data and quercetin concentration. Niacin (r = −0.313), vitamin B_6_ (r = −0.297), vitamin B_12_ (r = −0.266), vitamin D (r = −0.301), and ratio of animal protein to total protein (r = −0.27) were negatively correlated with quercetin concentration, whereas no components were positively correlated. FFQg data were correlated with fecal microbiota abundance.: *Sutterellaceae* (r = −0.292) and *Oscillospiraceae* (r = −0.334) were negatively correlated whereas *Fusobacteriaceae* (r = 0.361) and *Enterobacteriaceae* (r = 0.321) were positively correlated with quercetin concentration in the samples.

### Correlations among FFQg data, BMI, and fecal microbiota composition

Analysis of the relationship between BMI and FFQg data (intake of energy, and macronutrients and micronutrients) revealed weak negative correlations between BMI and intake of beta-carotene (r = −0.304) and beta-carotene equivalent (r = −0.291), and weak positive correlations between BMI and intake of Na (r = 0.278), Se (r = 0.354), niacin (r = 0.331), niacin equivalent (r = 0.283), vitamin B_12_ (r = 0.268), NaCl (r = 0.266), ethanol (r = 0.343), and alcohol (r = 0.273). Analysis of the relationship between BMI and fecal microbiota composition revealed weak negative correlations between BMI and *Porphyromonadaceae*, (r = −0.342), *Rikenellaceae* (r = −0.299), *Christensenellaceae* (r = −0.341), and *Oxalobacteraceae* (r = −0.329), as well as a weak positive correlation between BMI and *Aerococcaceae* (r = 0.32).

### Correlation between FFQg data and fecal microbiota composition

**Fig 3:**
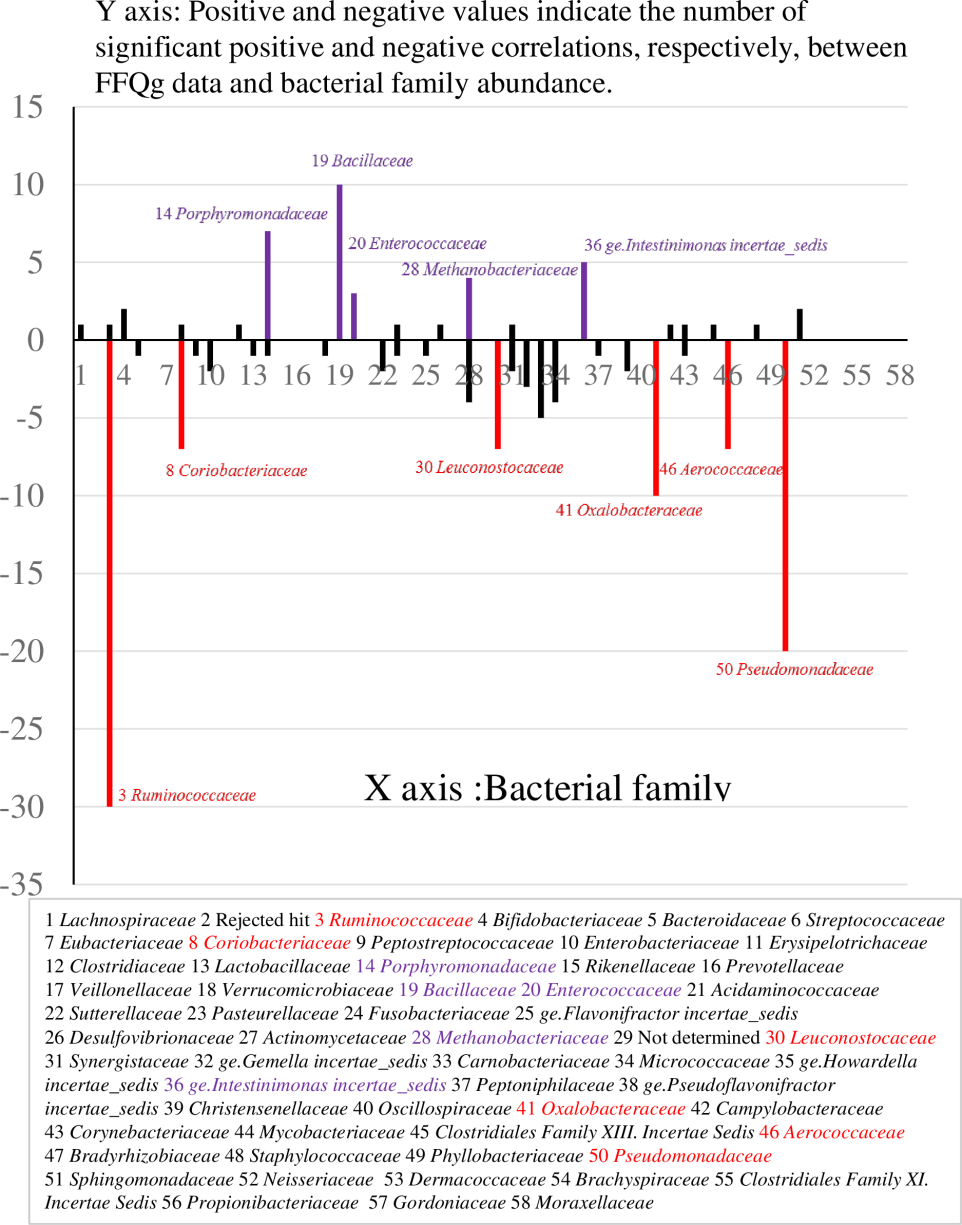
Correlations between FFQg data (intake of energy, and macronutrients and micronutrients) and fecal microbiota composition. Positive and negative values indicate the number of significant positive and negative correlations, respectively, between FFQg data (intake of energy, and macronutrients and micronutrients) and bacterial family abundance.Y axis: Positive and negative values indicate the number of significant positive and negative correlations. X axis: Bacterial family.

Significant correlations between FFQg data (intake of energy, and macronutrients and micronutrients) and fecal microbiota composition (occupation ratio of bacteria) are shown in Fig 3. The abundance of some bacterial groups was positively or negatively associated with the intake of specific food components in the FFQg data. *Ruminococcus* had the highest number of species (n = 30) that were negatively associated with FFQg data, followed by members of the *Pseudomonadaceae* family (n = 20). On the other hand, family *Bacillaceae* had the most taxons (n = 10) that showed a positive association with FFQg data, followed by *Porphyromonadaceae* (n = 7). *Methanobacteriaceae* had similar numbers of taxonomic groups showing positive and negative correlations with FFQg data (n = 4 each).

## Discussion

This is the first study to investigate the relationship between fecal quercetin metabolism and gut microbial community structure in healthy elderly subjects. The abundance of various bacterial families was positively or negatively correlated with quercetin metabolism, suggesting that the fate of quercetin in the lower gut depends on the composition of microbiota that metabolize this compound. Some intestinal bacteria degrade quercetin by anaerobic fermentation [20]. In the present study, intestinal bacteria metabolized most of the supplied quercetin in 24 h under anaerobic conditions despite inter-individual variations in fecal microbiota composition. Members of *Fusobacteriaceae* and *Enterobacteriaceae* are highly represented in the gut. Our results suggest that *Fusobacteriaceae* and *Enterobacteriaceae* affect quercetin bioavailability by directly or indirectly inhibiting the degradation of quercetin by other bacteria. The correlation analysis revealed that *Fusobacteriaceae* abundance was not significantly correlated with dietary intake; as such, it is unclear what type of diet can inhibit quercetin degradation. On the other hand, the abundance of *Enterobacteriaceae* was negatively correlated with vitamin D and B_12_ levels, which were negatively correlated with quercetin concentration after a 7-h incubation under anaerobic conditions. Thus, the quercetin degradation/*Enterobacteriaceae* occupation ratio may be increased by modifying diet.

Dietary quercetin and other polyphenols are absorbed by a small percentage (5–10%) in the small intestine and the rest of these molecules reaches the colon where they are metabolized by the gut microbiota, influencing its structure [21]. It has been reported that quercetin supplementation generated a great impact on gut microbiota composition [22] and dietary quercetin is supposed to exert potential prebiotic effect [23]. *Sutterellaceae* (r = −0.292) and *Oscillospiraceae* (r = −0.334) were negatively correlated with quercetin concentration in stool samples. *Sutterellaceae* and *Oscillospiraceae* may be related to quercetin's prebiotic effect. Further study is required to clarify the role of these taxa in quercetin metabolism.

An analysis of the relationship between FFQg data and fecal microbiota composition revealed bacteria that were positively or negatively correlated with the intake of specific food components. Bacteria whose abundance shows a low correlation with dietary intake may utilize short-chain fatty acids, host substances, or bacterial metabolites in order to survive in the gut.

Intestinal microbiota community structure differs between young and elderly subjects [24]. In general, diet affects community composition in the gut [25]; this as well as quercetin metabolism by microbiota can vary according to age.

*Christensenellaceae* abundance showed a weak negative correlation with BMI (−0.341). Low BMI has been linked to high *Christensenellaceae* levels in the human gut microbiome [26], while *Christensenellaceae*, *Mogibacteriaceae*, and *Rikenellaceae* were more abundant in lean (BMI < 25) as compared to obese (BMI > 30) subjects. *Christensenellaceae* may have BMI-lowering effects in the elderly [27]. In our study, *Porphyromonadaceae* and *Rikenellaceae* numbers were also found to be inversely related to BMI in this group.

It has been reported that dietary trans-10, cis-12-conjugated linoleic acid supplementation for 8 weeks significantly increased the proportions of Bacteroidetes, including *Porphyromonadaceae* bacteria and significantly decreased visceral fat mass (P< 0.001) [28]. *Coprobacter secundus* and *Alistipes inops* belong to the *Porphyromonadaceae* and *Rikenellaceae* families, respectively [29]. Both species produce acetic acid as metabolic end products [29], which plays an important role in lipid metabolism in mice on a high-fat diet by inducing the upregulation of genes encoding fatty acid oxidation enzymes and suppressing body fat accumulation [30]. Accordingly, pomegranate vinegar was shown to attenuate adiposity in obese rats [31]. Thus, members of these two taxa may modulate adiposity and contribute to health maintenance via production of acetic acid.

Intestinal microbiota can affect obesity [32], while diet can influence microbiota community structure [25]. Obesity is a metabolic syndrome; as such, clarifying the relationships among diet, obesity, and microbiota abundance is essential for disease prevention. The *Gemmiger*, *Dorea*, *Roseburia*, *Alistipes*, *Lactobacillus*, and *Bifidobacterium* genera were highly abundant in the gut microbiome of lean individuals [33]. In particular, *Bifidobacterium* has been negatively linked to obesity: *B*. *lactis* was associated with reduced obesity in patients with metabolic syndrome in a randomized trial [34]. On the other hand, *Ruminococcus bromii* and *R*. *obeum* are abundant in the gut of obese individuals [35]. In our study, the occupation ratios of Bifidobacteria and *Ruminococcaceae* were negatively correlated (r = −0.402). The occupation ratio of *Ruminococcaceae* was also negatively correlated with various food components, with a positive correlation observed only with cereal energy ratio. Thus, changes in the occupation ratio of *Ruminococcaceae* could affect that of Bifidobacteria, which can potentially be controlled by modifying food intake. However, since our research has a small number of samples, it will be necessary to further increase the number of samples to investigate the relationship between intestinal microbiota and BMI.

A limitation of this study was that we were unable to identify the type of diet required to reduce the degradation and thereby increase the bioavailability of quercetin. Nonetheless, our findings indicate that modifying diet can alter the gut microbiome and consequently quercetin metabolism, which can have health benefits in the elderly.

## Conclusions

This study investigated the relationship between quercetin metabolism, gut microbiota composition, and dietary intake in elderly Japanese 56 subjects. Inter-individual variations in quercetin concentration and fecal microbiota composition at family level suggested differences in microbial quercetin metabolism. The abundance of *Sutterellaceae* (r = −0.292) and *Oscillospiraceae* (r = −0.334) was negatively correlated whereas that of *Fusobacteriaceae* (r = 0.361) and *Enterobacteriaceae* (r = 0.321) was positively correlated with quercetin concentration. There were significant correlations between FFQg data and quercetin concentration. Analysis of the relationship between BMI and fecal microbiota composition revealed weak negative correlations between BMI and bacterial abundance. Bacterial abundance was positively or negatively related to intake of food components. This is the first report describing the relationship between fecal quercetin metabolism, human microbiota, and dietary intake in the elderly.

## Supporting information

S1 File **Data of quercetin concentration in fecal samples (A, B), fecal microbiota composition at the family level and the numbers of negative correlation and the numbers of positive correlation** (correlations between FFQg data and fecal microbiota composition).
(XLSX)

S1 Table Data of FFQg results. No1~No.56 indicate the volunteer number.
(XLSX)
